# Recent Developments in Lactone Monomers and Polymer Synthesis and Application

**DOI:** 10.3390/ma14112881

**Published:** 2021-05-27

**Authors:** Jakub Bińczak, Krzysztof Dziuba, Anna Chrobok

**Affiliations:** 1 Department of Chemical Organic Technology and Petrochemistry, PhD School, Silesian University of Technology, Akademicka 2a, 44-100 Gliwice, Poland; jakub.binczak@polsl.pl or; 2 Grupa Azoty Zakłady Azotowe, Puławy” S.A., Al. Tysiąclecia Państwa Polskiego 13, 24-110 Puławy, Poland; krzysztof.dziuba@grupaazoty.com; 3 Faculty of Chemistry, Silesian University of Technology, Krzywoustego 4, 44-100 Gliwice, Poland

**Keywords:** lactones, oligomers, polymers, application, synthesis

## Abstract

Lactones are a group of compounds that have been known for several decades. The commercial importance of lactones results from the possibility of manufacturing of a broad scope of derivatives and polymers with a wide spectrum of applications. In this work the synthesis and characterization of simple lactones are described, which due to the easy methods of the synthesis are of high importance for the industry. The chemical as well as biochemical methods are included with special attention paid to the methods that avoid metal catalysts, initiators or toxic solvents, allowing the use of the final products for the medical applications, e.g., for controlled drug-release systems, resorbable surgical threads, implants, tissue scaffolds or for the production of drugs. Lactone-based derivatives, such as polymers, copolymers, composites or three-dimensional structures are also presented. The work is focused on the methods for the synthesis of lactones and lactones derivates, as well as on the special properties and application of the studied compounds.

## 1. Introduction

Polyesters are very useful materials due to their biodegradability in the presence of enzymes, mechanical properties and many other important functional properties [1]. This significant potential of polyesters was a motivation for searching for the new structures of polyesters, while maintaining the biodegradability properties [2]. One of the useful monomers for polyester synthesis are lactones. Lactones were named by French chemist T-J. Pelouze who isolated the lactone as a derivative of lactic acid (LA) [3]. In 1880, German chemist W.R. Fittig extended the use of this name to all carboxylic esters which are obtained by an intramolecular reaction and are in the cyclic form [4]. In the systematic nomenclature, lactones form the group of 1-oxacycloalkan-2-ones, while the bulk of their names are common names derived from carboxylic acids with the alkyl chain length indicated. Additionally, a Greek letter as a prefix is used to indicate to which carbon the oxygen of the carboxyl group is attached, e.g., the four-membered ring lactone contains the Greek letter β.

**Scheme 1:**
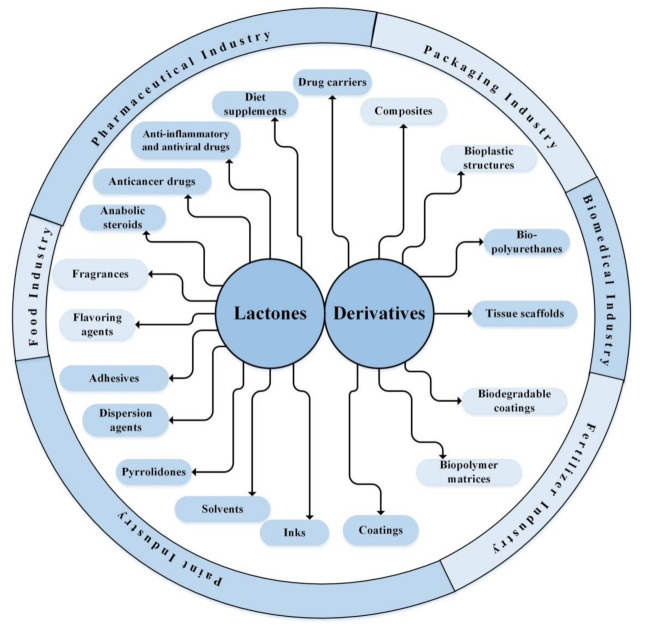
Versatility of use of lactones and their derivatives in the industry.

Lactones and their derivatives are used in many areas of the modern chemical industry (Scheme 1). Lactone-based polymers and copolymers, due to their biodegradability and biocompatibility, are mainly used in the medical industry, as scaffolds for the regeneration of bones and soft tissues, as well as in the pharmaceutical industry as the carriers of the active ingredients [5]. However, due to the strict regulations concerning the sustainable use of plastics, lactone-based polymers and copolymers are gaining importance in the packaging industry [6]. Lactone copolymers can also be used in the fertilizer industry as coatings for fertilizers with slow and/or controlled release of nutrients, which in turn will help to reduce the leaching of these components to the soil and atmosphere [7]. In addition, composites, nanocomposites and coated biostructures are noteworthy, since they add to the polymers and copolymers’ additional properties, such as the rigidity of the three-dimensional structure or antibacterial properties [6]. There are currently several reviews concerning lactones and their polymerization [8,9,10]. This work includes not only an overview of their recent preparation and polymerization possibilities, but also properties of their new polymers/copolymers and recent application directions.

## 2. Properties of Lactones

Generally, lactones with a simple structure are used as monomers, precursors of biodegradable polymer materials. Lactones with a more complex, polycyclic structure (macrolactones) are used in medicine, as anti-inflammatory or anti-malarial compounds as well as bioactive compounds against various diseases, cancer cells, parasites, viruses, bacteria and anabolic steroids or synthetic musks. The helenalin (sesquiterpene lactone) is an example of a lactone with anti-inflammatory properties which also exhibits antiparasitic activity. An artemisinin which is active against malaria can be another example. An alantolactone, epothilone or actinomycins comprising lactone moieties are useful in the treatment of tumours. Antibacterial activity is demonstrated by, among others, xanthatin and tavulin, and dentin and tannachine. Polycyclic systems with an α-methylene-γ-butyrolactone ring, which include centaurepressin or chlorojanerine, are characterized by antiviral activity. Anabolic properties are exhibited by oxandrolone, while ω-pentadecalactone can be used as synthetic musk [11].

**Scheme 2:**
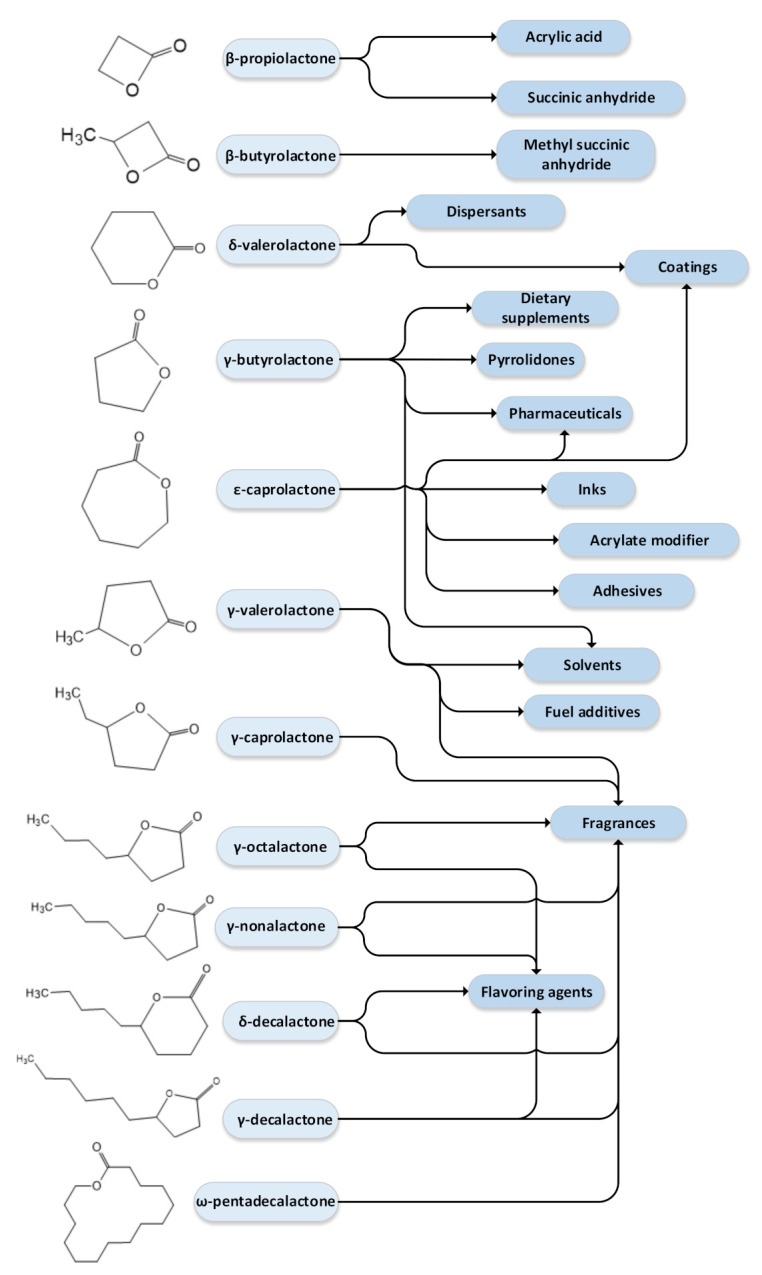
Versatility of the use of lactones and their derivatives’ products in the industry.

β-propiolactone offered *i.a.* by Novomer Inc. is one of the simplest lactones and is useful in the production of acrylic acid, acrylic esters and succinic anhydride, and also, as a monomer or comonomer in the production of biodegradable polymeric materials [12]. Simple lactones such as δ-valerolactone, ε-caprolactone or γ-butyrolactone are commercial products offered by BASF. δ-valerolactone is used as an intermediate in the production of coatings, dispersants and as a comonomer for polymerization with ε-caprolactone, which is responsible for the lowering of the melting point of the resulting copolymer or oligomer [13]. Additionally, δ-valerolactone is used for the preparation of the homopolymer poly-δ-valerolactone. γ-valerolactone can be used both as a monomer in the production of polymer, but also directly as a fuel additive, fragrance or green solvent. Furthermore, ε-caprolactone is mainly used as an intermediate in the production of a number of polymeric structures which, depending on the needs, include hydroxyl or carboxyl groups as the end groups. It is used, among other things, in the production of polyurethane dispersion (PUD) coatings, adhesives, inks or pharmaceuticals. ε- caprolactone is also used as a modifier of acrylate or methacrylate monomers, such as hydroxyethylmethacrylate, hydroxyethylacrylate, hydroxypropylacrylate or glacial methacrylic acid [14]. ε-caprolactone-modified acrylate monomers, due to the shifting of the hydroxyl group from the main alkyl chain of acrylic resins, are used together with cross-linking monomers [13]. The spectrum of the application of ε-caprolactone and other commercially available lactones is presented in Scheme 2.

γ- and β-butyrolactones are of wide industrial importance, due to an array of possible applications. Butyrolactone monomer is used as a solvent in paints, nail polish removers, printed circuit board (PCB) cleaners and as an intermediate in the production of pyrrolidones. It is present also in the production of pharmaceuticals, and in the USA it was sold as a dietary supplement which was used to support falling asleep, releasing growth hormones and improving physical conditions, but no therapeutic effect of this lactone has been confirmed [15].

γ-butyrolactone is produced by Mitsubishi Chemical Corporation and can also be used to produce a selected hydroxy-γ-butyrolactone enantiomer. The β-butyrolactone can be used, like most lactones, for the production of its homo and copolymers, but it is also used in the reaction with carbon monoxide, for the stereoselective production of methyl succinic anhydride [16]. Lactones derived from carboxylic acids with longer alkyl chains, such as γ-octalactone or γ and δ-decalactones, can be used as flavours or fragrances [17].

## 3. Methods for the Synthesis of Lactones

**Scheme 3:**
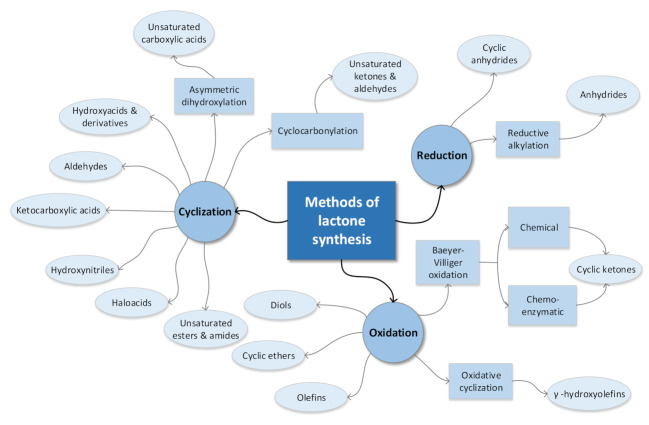
The main methods for the synthesis of lactones.

The methods for the preparation of lactones differ depending on the character of the product: a macrocyclic or a simple lactone. A chemical synthesis of macrolactones is complicated, characterized by multistep reactions, and difficult to apply in the industry. Other common methods include the Corey–Nicolaou, Boden–Keck or Mitsunobu macrolactonisations [18]. The most popular methods include the Yamaguchi esterification reaction and Shiina macrolactonisation. The synthesis of the simple lactones using chemical methods can be divided into cyclization, reduction and oxidation. In recent years, biocatalysts were also used for the synthesis of lactones, enabling the use of less harmful and more stable reagents [19]. The main methods for the preparation of lactones are presented in Scheme 3.

**Figure 1:**
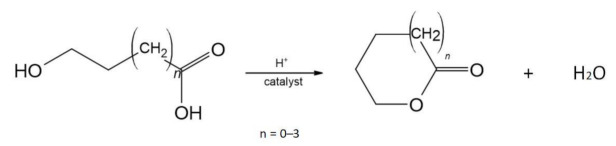
General scheme of the hydroxyacid cyclization reaction.

One of the most popular methods for the synthesis of lactones is the highly efficient cyclization of hydroxyacids and their derivatives which is catalysed by acids (Figure 1). Inorganic acids as well as sulfonic acid, p-toluenesulfonic acid and pyridine p-toluenesulfonate are the most commonly used catalysts [20]. Another synthetic path for the synthesis of δ-lactones is the oxidation (dehydrogenation) of diols and δ-hydroxyaldehydes. In recent years, BASF has developed new catalysts which are transition metals supported on activated carbon and inorganic oxides of aluminium, silicon, zinc, titanium, iron, chromium or zircon, useful in the dehydrogenation of 1,5-pentadiol [21].

The γ-valerolactone can be obtained by the cyclization of levulinic acid which belongs to the group of γ-ketocarboxylic acids. This method is based on the use of hydrogen, possibly sourced from the electrolysis of water, and the reaction is carried out using catalysts based on noble metals, of which ruthenium catalysts show the highest catalytic activity. The dehydration of levulinic acid to α-angelica lactone followed by the hydrogenation of the latter to γ-valerolactone is also known. The second step can be performed using immobilized lipase B from *Candida antarctica* (CALB) [22]. Novomer Inc. proposed the synthesis of β-propiolactone using ethylene oxide and waste carbon monoxide [23]. This method can be classified as the oxidation of cyclic ethers. On the other hand, the synthesis of γ-butyrolactone may be carried out by the previously described cyclisation of a hydroxyacid by dehydration of γ-butyric acid [15]. The method, used mainly by BASF, involves a series of dehydrogenation reactions starting from 1,4-butanediol, through γ-hydroxybutyroaldehyde and 2-hydroxytetrahydrofuran to γ-butyrolactone. The process is carried out in the presence of a copper catalyst at 180–300 °C and the by-product is hydrogen which, after purification and removal of the residual carbon monoxide, for example by methanation, can be recycled back to the process [24].

The method used by Mitsubishi Chemical Corporation is based on the hydrogenation of maleic anhydride at 160–280 °C and a pressure of 6–12 MPa in the presence of a nickel catalyst. A by-product of this reaction is tetrahydrofuran, the formation of which can be controlled by the reaction conditions [25]. A more complicated method, with maleic anhydride and methanol as the main raw materials, is used by Johnson Matthey Davy Technologies [26].

**Figure 2:**
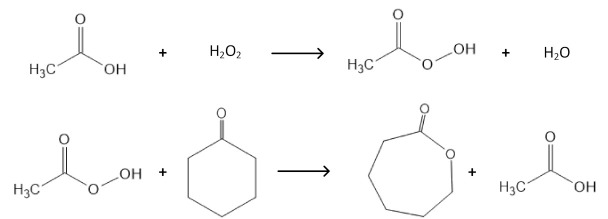
The synthesis of ε-caprolactone using the Perstorp method.

The manufacturing process of β-butyrolactone was patented in the early 1950s by the U.S. corporation Union Carbide and Carbon Co., now belonging to the Dow Chemical Company. It is based on the use of activated montmorillonite, ethyl ether, gas-phase ketene and acetaldehyde [27]. One of the most useful and studied reactions for the preparation of lactones is the Baeyer–Villiger oxidation reaction [28,29,30]. ε-caprolactone is obtained mainly in the Baeyer–Villiger oxidation of cyclohexanone. The most popular method so far, used by the Perstorp company, involves the use of peracetic acid as the oxidant, which is obtained in the first stage by the oxidation of acetic acid with hydrogen peroxide (Figure 2). During the oxidation of cyclohexanone, peracetic acid is reduced back to acetic acid and is recycled to the first stage. The unreacted cyclohexanone is separated in the distillation and recycled to the second reaction stage. The high reactivity of peracetic acid lowers the safety of the process [31].

**Figure 3:**
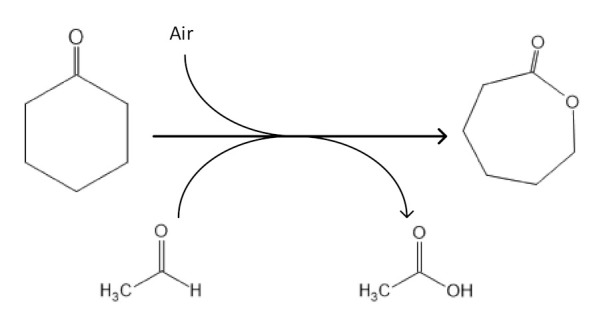
The synthesis of ε-caprolactone using the BASF method.

In an alternative process proposed by BASF, the oxidation of cyclohexanone is carried out using acetaldehyde in an air atmosphere (Figure 3). The advantage of this method is the reduction of the step requiring the transfer and contact with the unstable peracetic acid, due to the one-pot procedure. The disadvantage of this process is the lack of the easy possibility of the acetic acid recycling [32].

An interesting alternative is the process in which perdecanoic acid is used as an oxidant. Perdecanoic acid is characterized by much higher stability than acetic acid and the by-product decanoic acid can be returned to the percarboxylic acid formation stage. This significantly improves the safety of the entire process and, crucially, reduces the amounts of generated wastes [33].

**Scheme 4:**
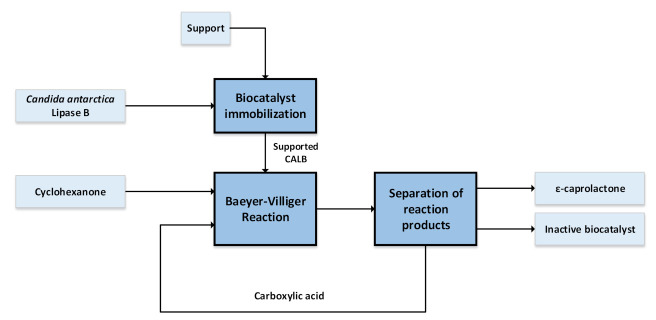
Schematic diagram of the chemo-enzymatic method of ε-caprolactone synthesis.

**Figure 4:**
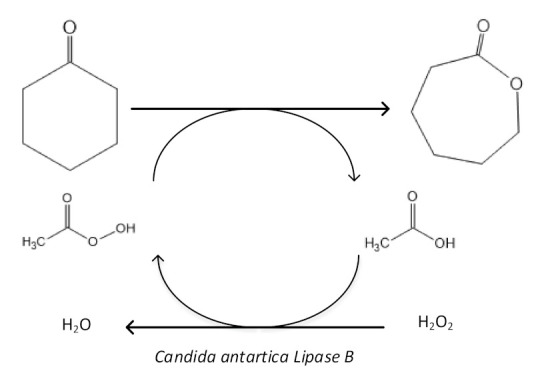
The synthesis of ε-caprolactone using the chemo-enzymatic method.

Special attention should be paid to the chemo-enzymatic oxidation of cyclohexanone to ε-caprolactone, distinguished by the use of a heterogeneous biocatalyst CALB supported on the multi-wall carbon nanotubes. The use of the enzyme in this method allows the Baeyer–Villiger oxidation to be carried out in one step using medium to long chain carboxylic acid and hydrogen peroxide (Figure 4). The proper oxidant, percarboxylic acid, is formed in situ and immediately oxidizes the cyclohexanone present in the reaction medium while the carboxylic acid is once again oxidised in the reaction mixture. The reaction takes place under the mild conditions (Scheme 4) [19].

ε-caprolactone can be obtained in the presence of acidic ionic liquids as a catalyst and a urea complex with hydrogen peroxide as the oxidant. The yield of this method towards the main product reached 91% [34]. A microbiological method of the γ and δ-lactones synthesis, from carboxylic acids and their derivatives with chain lengths from C4 to even C21 with the use of Mucor fungi from the Mycoses, is also known and was patented over 30 years ago by the BASF company. γ-octalactone, γ-caprolactone, δ-caprolactone and γ-decalactone were fabricated in 5–20 L bioreactors using this method [35].

## 4. Products Produced from Lactones and Their Applications

**Scheme 5:**
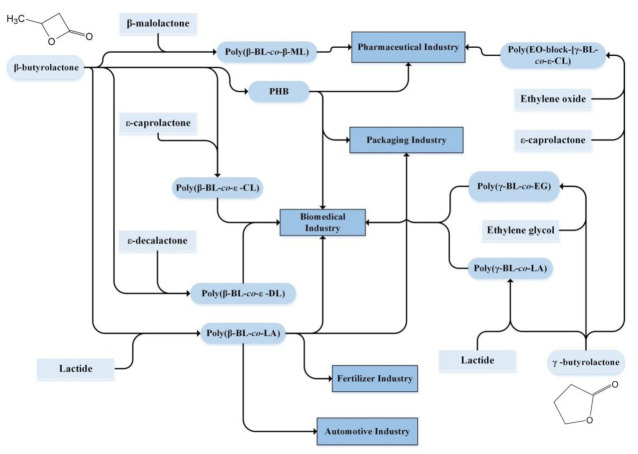
Products produced from butyrolactones and their areas of application.

Polymers or copolymers-based lactones are used in many sectors of the industry; however, not all of them can be successfully used considering the toxicity, e.g., β-propiolactone-based products. [36]. As mentioned earlier, polymers based on lactones can be used in medicine as bioresorbable materials. Some of them can be widely used in medicine due to the approval by the FDA. These include, in particular, PCL and Polylactic acid (PLA) [37,38]. Their usefulness in a particular application depends on their mechanical and physical properties. Copolymerization is the method to adjust these properties to obtain the desired parameters, such as biodegradation time, rigidity and thermal properties. However, the solvents and initiators used for the preparation of polymers and copolymers should be particularly carefully chosen. Many conventional methods use tin catalysts; yet, their toxicity precludes the use of the reaction products in medicine [39]. The polymers and copolymers made from butyrolactones and their application examples are shown in Scheme 5.

**Scheme 6:**
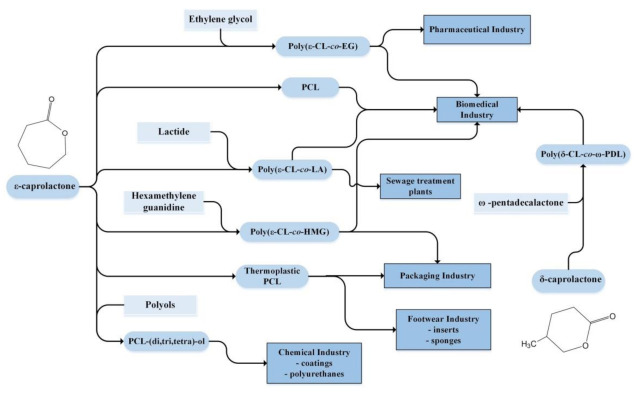
Products obtained from caprolactones and their possible applications.

One of the most popular products coming from β-butyrolactone is polyhydroxybutyrate (PHB). It belongs to the group of polyhydroxyalkanoates, which are future “green” polymeric materials for biomedical applications, packaging and controlled drug release systems [40]. Unfortunately, it has poor mechanical properties due to its fragility and tendency toward thermal degradation. Bioresorbable applications use block copolymers of β-butyrolactone as the soft segment and ε-caprolactone, lactide (LA) or ε-decalactone as the segment improving mechanical and thermal properties [41,42,43,44]. Copolymerization of β-butyrolactone with β-malolactone allows the obtaining of structures with biologically active centres [45]. An improvement of the mechanical properties of γ-butyrolactone can be obtained by its copolymerization with ethylene glycol. The solubility in water of the final product increases as well as the biodegradation rate and biocompatibility [46]. In tissue engineering, a copolymer of γ-butyrolactone with LA may be used [47]. Interesting applications in the drug delivery systems are block copolymers of γ-butyrolactone, ε-caprolactone and ethylene oxide. Their amphiphilicity obtains micellar structures which in the centre of the hydrophilic structures contain closed, hydrophobic micelles with an active ingredient. The created structure could ensure an appropriate release profile of the active ingredient, protecting it against premature interaction with the organism [48]. Polymers of δ-valerolactone exhibit properties similar to poly-ε-caprolactone (PCL) due to the similar structure. They are less flexible but, as in the case of ε-caprolactone, their polymerization with LA can give biocompatible products suitable for the regeneration of the soft tissue or construction of implants. Copolymers of LA with lactones such as ε-caprolactone or δ-valerolactone can serve as carriers for nutrients in agriculture. Impregnation with, for example, repellents allows for a product that during biodegradation is characterized by a slow release of the ingredient, which positively affects the sustainability of agriculture and reduces its negative impact on the environment [49]. PCL, during the breakthrough into resorbable polymers in the 1970s and 1980s, was mainly used in the field of biomaterials and drug delivery systems due to its mechanical properties, biodegradability and compatibility with other polymers. Its rheological properties make it easy to manufacture and process into implants [50]. The possible direct use of polymers and copolymers of caprolactones are shown in Scheme 6.

**Scheme 7:**
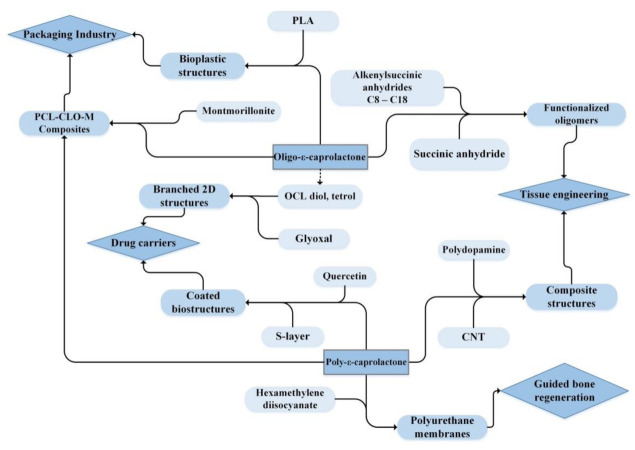
The application of ε-caprolactone in the synthesis of the modern materials.

The biodegradation rate of PCL depends on the content of the crystalline phase of the polymer. By lowering the ratio of the crystalline to the amorphous phase, for example by copolymerization, the increasing of biodegradation and bioresorption rates is observed. The copolymerization of ε-caprolactone with ethylene glycol allows the use of the copolymer inside the human body, e.g., implants and tissue regeneration, and also as carriers for drugs with controlled release [51]. Copolymers of ε-caprolactone with LA can also find the application in the purification of water from organic pollutants. In recent years, biodegradable polymers with antibacterial properties have become popular both in medical applications and in the packaging industry. Such bifunctionality is demonstrated by a copolymer of hexamethylene guanidine with ε-caprolactone, which combines the biodegradable properties of ε-caprolactone with the antibacterial properties of hexamethylene guanidine [52]. PCL is also used not only in copolymers but also as a standalone modified polymeric material. Scheme 7 shows the application of ε-caprolactone polymers in the production of the modern polymeric materials, biocompatible mixtures and nanocomposites.

Composites made of biodegradable polyesters and layered silicates, e.g., PCL in the combination with oligo-ε-caprolactone (OCL) and the addition of montmorillonite, are not only environmentally friendly but also inexpensive to manufacture due to the use of natural minerals. The resulting composites are characterized by both biodegradable properties resulting from the use of polyesters and better mechanical properties, compared to pure polymer, owing to the use of a mineral component. This characteristic applies the obtained composites in the packaging industry [53]. Functionalization of PCL and OCL end groups allows for the creation of two and three-dimensional structures, which, depending on the needs, allow the control of the rate of biodegradation and the thermal and mechanical properties. An example of such structures is OCL terminated with hydroxyl groups which are cross-linked with glyoxal. Such cross-linked structures may have temperature-sensitive points, which make them suitable for shape memory materials. Permanently cross-linked, functionalized OCL can also be used in tissue regeneration and in the printing industry [54]. Mixtures of PCL with flavonoids that have antioxidant properties can be used as drug carriers [55]. Polyurethane membranes based on PCL can be used in bone regeneration supported by skeletal membranes. Due to the biodegradability of these materials, after the bone tissue regeneration process there is no need to perform an additional surgical operation to remove the skeleton supporting the regeneration [56]. In the field of tissue engineering, nanocomposites and PCL-based nanofibers formed by electrospinning play a significant role. Carbon nanotubes serve as tissue scaffolds, while the addition of biologically active compounds, such as polydopamine (PDA), improves the adhesion of cells and thus their regeneration [57]. The Japanese company Daicel Chemicals offers a number of products based on ε-caprolactone, *i.a.* ε-caprolactone-modified epoxy carboxylates which belong to the group of alicyclic tetra epoxides and are used as cationic coatings. PCL diols, triols and tetraols that are used in the preparation of polyurethane coatings have been gaining interest as well [58]. The ε-caprolactone polyols are also manufactured by Perstorp which was acquired by Ingevity Corporation. They are used in the film-forming resin industry as polyurethane modifiers but also as cast elastomers. The company also produces ε-caprolactone-based thermoplastics for the packaging industry, orthopedic insoles, shoe sponges and adhesives [59]. As an alternative to the PCL for medical use, a copolymer of ω-pentadecalactone with δ-caprolactone is also proposed [60]. Poly δ-decalactone with a relatively long, strongly hydrophobic alkyl chain allows for the application of larger loads of the active ingredient in comparison to the polymers currently used [61]. The packaging industry expects materials with mechanical properties similar to these which are used presently but, unlike them, are also biodegradable. One such substitute may be poly ω-pentadecalactone, with its similar strength and mechanical properties to low density polyethylene. The control over the mechanical and thermal properties can be ensured by a combination of both ε-decalactone and LA, as well as ε-caprolactone in the form of a ternary elastomer [62]. The amorphous nature of poly-ε-decalactone also contributes to the increasing medically active ingredient loads. Thanks to this, ε-decalactone is an excellent comonomer, for example, in combination with methoxy-ethylene glycol, for the production of micellar drug carriers [63].

## 5. Methods for the Synthesis of Lactones Derivatives

The development of the new techniques for the synthesis of state-of-the-art copolymers and polymeric materials with the use of lactones was a subject of many studies in the last years [36,37,38,39,40,41,42,43,44,45,46,47,48,49,50,51,52,53,54,55,56,57,58,59,60,61,62,63,64,65,66,67,68,69,70,71,72,73,74,75,76,77,78,79,80,81,82,83,84,85,86,87,88,89,90,91,92,93,94,95,96,97,98,99,100,101,102,103,104,105,106,107]. The main trend in their synthesis is the search for new, non-toxic and cheap catalysts and initiators for polymerization and copolymerization reactions. Table 1 lists the initiators or catalysts used in some of the polymer/copolymer preparation methods based on the known lactones.

**Scheme 8:**
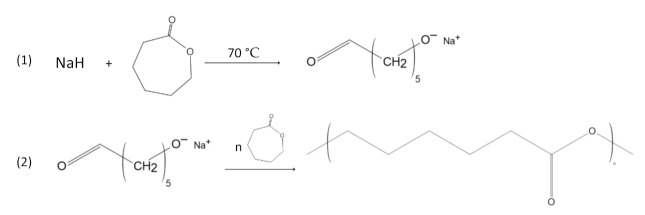
Preparation of a PCL using NaH as an initiator.

As mentioned in the previous section, the interest in research and the possibilities of using polymers and copolymers of β-propiolactone have decreased in recent years due to its potential carcinogenic properties [36]. As a result, these compounds were practically excluded from use, both in the biomedical industry, as well as in products that have long-term contact with humans. The methods of copolymers synthesis in most cases are used for the copolymerization of comonomers. However, most of the developed methods use catalysts or initiators based on heavy metals or toxic organic compounds. Therefore, methods that use compounds that are completely safe for the environment are of exceptional importance. In the preparation of the copolymer of β-butyrolactone with ε-caprolactone, ring-opening polymerization is proposed using NaH as the initiator [41]. The β-butyrolactone is polymerized to polyhydroxybutyrate (PHB) in ring-opening polymerization using the above-mentioned NaH as an initiator. The anionic homopolymerization of ε-caprolactone is also possible using this initiator (Scheme 8) [44].

**Scheme 9:**
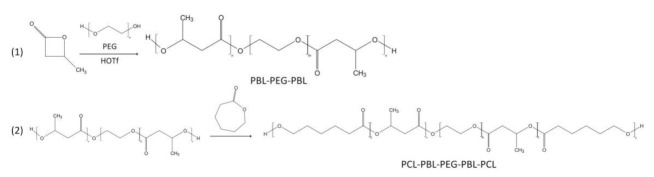
Preparation of a poly(ε-CL-*co*-β -BL-*co*-EG) block copolymer.

The preparation of three-component block copolymers of β-butyrolactone, ε-caprolactone and ethylene glycol is carried out with the trifluoromethanesulfonic acid as a catalyst. At the first stage of the process, the synthesis of a two-component block copolymer of β-butyrolactone and ethylene glycol is conducted, with the polyethylene glycol serving as a macroinitiator. At the next stage, ε-caprolactone is introduced into the reaction mixture, which results in the formation of a ternary block copolymer (Scheme 9) [74].

**Scheme 10:**
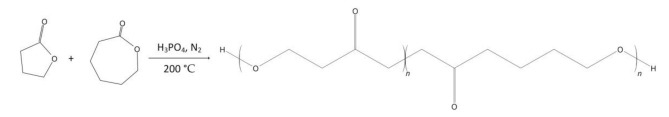
Preparation of a poly(γ-BL-*co*-ε-CL) copolymer.

Polymerization of γ-butyrolactone to poly-γ-butyrolactone may be performed using zeolites as the catalysts. This is especially interesting from the point of view of sustainable development and the search for environmentally friendly production methods. It has been found that by running the reaction at 180 °C for about 6 h, a 47% of oligomer can be obtained [76]. An interesting product is a three-component block copolymer of γ-butyrolactone, ε-caprolactone and ethylene oxide. The reaction is carried out for 24 h under argon atmosphere, using 1,5,7-triazabicyclo [4.4.0] dec-5-ene (TBD) as the catalyst [48]. The preparation of a copolymer of γ-butyrolactone and ethylene glycol may be carried out using an organophosphorus catalyst, N-(4-Chloro-3-trifluoromethyl-phenyl) -2-ethoxy-6-pentadecyl-benzamide (CTPB) and methyl ethers of poly(ethylene glycol) as initiators [46]. In a similar manner, using the same catalyst, ring-opening copolymerization can be performed with γ-butyrolactone and LA [47]. Both methods described above avoid metallic initiators. Valerolactones can undergo copolymerization with other lactones, such as ε-caprolactone, but also with LA or ethylene glycol. Metal catalysts and activators are important in these reactions. The copolymerization of γ-valerolactone with ε-caprolactone can also be carried out without the presence of a catalyst, under nitrogen and in a dilute phosphoric acid medium. In the same manner, the copolymerization of γ-butyrolactone and ε-caprolactone can be performed (Scheme 10) [82].

A method of oligomerization of δ-valerolactone using initiators in the form of heteropolyacids (HPA) is also known. It has been proven that in the case of using the HPA-2 heteropolyacid (H_5_[PMo_12_−2V_2_O_40_]·aq/MeOH) linear oligomers are obtained [85]. The synthesis of ε-caprolactone copolymers with various compounds, e.g., other lactones (ω-pentadecalactone, γ-butyrolactone or γ-valerolactone), carboxylic acids, methyl esters, glucopyranosides and carbonates was studied [90,91,92,93,94,95,96,97,98,99,100]. Popular comonomers with caprolactone also include LA and ethylene glycol. Like most of the reactions discussed above, the copolymerization of δ-caprolactone with ω-pentadecalactone is relatively simple and is based on a “one pot” ring opening polymerization. It runs under a nitrogen atmosphere, at 130 °C for 6 days with triphenyl bismuth as a catalyst but without the addition of an initiator. By reducing the amount of the catalyst the molecular weight can be lowered [60]. Attention is also drawn to the possibility to copolymerize ε-caprolactone with γ-butyrolactone and γ-caprolactone without the addition of a catalyst, in a dilute phosphoric acid medium. However, the lack of a component able to reduce the energy of activation influenced the increasing of the temperature to 200 °C and a relatively long reaction time of up to 3 days [82]. Recently, interest has also been raised by the copolymerization of ε-caprolactone with carbonates. Copolymerization of ε-caprolactone in trimethylene carbonate can be performed using methanesulfonic acid as a catalyst [92]. Interesting materials with antibacterial properties include a copolymer of ε-caprolactone and polyhexamethylene guanidine. The reaction is carried out at 160 °C with polyhexamethylene guanidine as a macro initiator yielding 94% of the product with molar weight depending on the molar ratio of the reactants [52]. Over the last few years, methods of polymerization and copolymerization of lactones using biocatalysts of natural origin have been of particular interest. Enzymatic methods with CALB are very attractive, e.g., for the copolymerization of 5-hydroxymethyl-2-furancarboxylic acid and ε-caprolactone in a “one-pot” reaction at 80 °C [100]. In a similar process, using immobilized CALB, oligoesters of ε-caprolactone and glucopyranoside can be obtained. The reaction is carried out at 80 °C and the conversion of ε-caprolactone after 48 h reached 90%. The molecular masses of the fabricated oligoesters are within 1600 g/mol [97]. The earlier described "one-pot" preparation of ε-caprolactone with cyclohexanone using an immobilized CALB, hydrogen peroxide and a carboxylic acid can also be used to synthesize the oligomer. The lack of an oligomerization inhibitor results in OCL synthesis, thus the transition from the substrates, i.e., cyclohexanone and hydrogen peroxide to the oligomer itself, actually takes place in one vessel. Moreover, this method is characterized by mild reaction conditions (low temperature and atmospheric pressure) and the absence of environmentally harmful initiators or catalysts containing heavy metals [19].

Lactone homopolymers, e.g., PCL, are functionalized with other compounds, such as succinic anhydrides or structures such as carbon nanotubes; however, they are also used for the production of mixtures, nanocomposites or as biodegradable plasticizers [53,54,55,56,57]. Methods for the synthesis of PCL-based products are described below. The methods of preparing polymer or oligomer blends with an additional component are relatively simple and are based on mixing both components in a solvent and then applying this solution onto a desired material or simply a plain surface. After the evaporating of the solvent, a blend-covered product or a thin film is obtained [98]. Diols of OCL, and also triols and tetraols, are suitable for the production of two-dimensional structures. The chain terminated with hydroxyl groups is functionalized. Interesting complex structures can be obtained especially with the use of triols and tetraols. An example of this is the formation of hemiacetals in the reaction of OCL tetraol with glyoxal, which serves as a cross-linker, with the application of a method for creating Langmuir films on the surface of an aqueous solution [54]. OCL is functionalized in a similar way with succinic and alkenesuccinic anhydrides [107]. A method of the synthesis of OCL and PCL nanocomposites with montmorillonite is also important. In the first step, functionalization of OCL with 2-dibutylamino-ethanol is performed. Next, the ammonium-terminated OCL undergo ion-exchange with montmorillonite, and the arising "organic clay" is mixed with PCL [53].

It is possible to obtain PCL/CNT fibres using electrospinning methods. PCL structures can also be modified with PDA. The deposition of PDA on the surface of PCL structures is affected relatively simply by rinsing PCL mats with a solution of PDA in hydrochloric acid [57]. Polyurethane materials based on PCL can be obtained in a two-step process. In the first step, a PCL diol is added to a hexamethylene diisocyanate solution. In the second step, synthesis of three-component polyurethane blocks is carried out by adding polyethylene glycol or ethylenediamine [56].

Decalactone derivatives products are obtained in a similar way to the previously described lactones. Polymers of δ-decalactone and ε-decalactone alone, but also their copolymers with LA, other lactones such as as ω-pentadecalactone, ε-caprolactone or glycols such as the polyethylene glycol and methoxy polyethylene glycol are crucial products. Attention is drawn to the possibility of the homopolymerization of ε-decalactone using indium compounds. Indium chloride can also be used as a catalyst in the block copolymerization of ε-decalactone with ε-caprolactone [105]. The copolymerization of ε-decalactone with ω-pentadecalactone can be performed in the “one pot” synthesis type. Both monomers are added simultaneously to the reactor in a nitrogen atmosphere, and the catalyst in the form of triphenyl bismuth is also added. This method does not use any initiator [103].

## 6. Conclusions

Lactones constitute a wide group of cyclic esters that have been known for over 150 years, contributing to industrial applications such as fuel additives, modifiers, inks and dietary supplements as well as flavours or fragrances. However, their most important impact is visible in the production of biocompatible and biodegradable polymers.

Various methods of lactone production were developed, which can be generally divided into cyclization, reduction and oxidation reactions. Especially worth noting are recently developed methods of oxidation of cyclic ketones with the use of biocatalysts. Their competitive advantage consists not only in using environmentally friendly catalysts but also, in the "one-pot" type synthesis, which in turn increases the safety of the process. Recently, multiple studies were directed into the synthesis of polymers and copolymers of lactones. The mechanical and thermal properties of the resulting product depend on the lactone. The vast majority of methods for the synthesis of derivatives involve organic tin (II) compounds as catalysts. On the other hand, methods using catalysts and initiators free of heavy metals or derived from the natural sources such as zeolite or minerals are of great potential. The most common polymerization and copolymerization reaction mechanism is ring-opening polymerization. The amount of publications and research in recent years on the production and possible use of lactone-derived products indicates substantial interest in these materials and, undoubtedly, their rising market importance. This fact in parallel with the ongoing changes in legislation push the economy towards sustainable development and promote the use of biodegradable materials.

## Figures and Tables

**Table 1:** Products obtained from the polymerization and copolymerization of lactones.

Monomer	Comonomer(s)	Product	Initiator or Catalyst	Source
β-propiolactone	Valine	Poly(VAL-*co*-β-PL)	NaOH/MeOH	[64]
	–	Poly-β-propiolactone	Temperature	[65,66]
			FeCl_3_	[65]
			H_2_ SO_4_	
	γ-butyrolactone	Poly(γ-BL-*co*-β-PL)	AlEt_3_-H_2_ O	[67]
	Lactide	Poly(LA-*co*-β-PL)	TfOH/MeOH	[68]
	Glycolide	Poly(G-*co*-β-PL)	Anionic catalyst	[69]
			Acidic catalyst	
			Complexing catalyst	
	Acetonitrile	Poly(AN-*co*-β-PL)	α, α’-azobisisobutyronitrile benzoylperoxide	[70]
	Styrene	Poly(S-*co*-β-PL)		
	2-oxazoline	Poly(OXZ-*co*-β-PL)	Temperature	[71]
	PEG	Poly(EG-*co*-β-PL)	Temperature	[72]
β-butyrolactone	ε-caprolactone	Poly(β-BL-*co*-ε-CL)	NaH	[41]
	–	PHB	NaH	[44]
			Sodium phenoxides	[40]
			Aluminum complexes	[73]
	ε-decalactone	Poly(β-BL-*co*-ε-DL)	Y (III) complexes	[42]
	β-malolactone	Poly(β-BL-*co*-β-ML)	Metal catalyst/Isopropanol	[45]
	Lactide	Poly(LA-*co*-β-BL)	Y (III) complexes	[43]
	ε-caprolactone, PEG	Poly(ε-CL-*co*-β-BL-*co*-EG)	Trifluoromethanesulfonate sulfone acid	[74]
	γ-valerolactone	Poly(β-BL-*co*-γ-VL)	BF_3_-OEt2	[75]
γ-butyrolactone	ε-caprolactone, Ethylene oxide	Poly(EO-*co*-ε-CL-*co*- γ-BL)	TBD	[48]
	–	Poly-γ-butyrolactone	Zeolites	[76]
	PEG	Poly(EG-*co*-γ-BL)	CTPB/m PEG	[46]
	Lactide	Poly(LA-*co*-γ-BL)	CTPB	[47]
α-methylene-γ-butyrolactone	–	PMBL	Cu/( photoATRP )	[77]
			Benzyl alcohol/urea	[78]
	Styrene	Poly(S-*co*-α-M-γ-BL)	AIBN	[79]
δ-valerolactone	ε-caprolactone	Poly(δ-VL-*co*-ε-CL)	Pyridyl urea/MTBD	[80]
			Zinc 2,6-bisaminophenol complex	[81]
			Temperature	[82]
	Lactide	Poly(LA-*co*-δ-VL)	Bismuth sub-sallycinate (III) ( BiSS )	[83]
			Tin octanoate (SnOct_2_ )	[49,83]
	ε-caprolactone,PEG	Poly(δ-VL-*co*-ε-CL- *co*- EG)	ITU/YCl_3_ (2,3,6,7-tetrahydro- 5H-thiazolo [3,2-a] pyrimidine)	[84]
	–	Oligo(δ-VL)	H_5_ [PMo_12_−2V_2_ O_40_ ] · aq / MeOH	[85]
γ-valerolactone	ε-caprolactone, mPEG	Poly(mEG-*co*-ε-CL- *co*-γ-VL)	Tin octanoate (SnOct_2_ )	[86]
	ε-caprolactone	Poly(ε-CL-*co*-γ-VL)	mPEG/octanoate, tin (SnOct_2_)	[87]
			Temperature	[82]
δ-caprolactone	ω-pentadecalactone	Poly(ω-PDL-*co*-δ-CL)	Triphenyl bismuth (Ph_3_Bi)	[60]
	–	Poly-δ-caprolactone	TBD/Benzyl Alcohol	[88]
ε-caprolactone	–	Poly-ε-caprolactone	4-dimethylaminopyridine (DMAP), Lewis acid/Benzyl alcohol	[89]
	γ-butyrolactone	Poly(ε-CL-*co*-γ-BL)	Heat	[82]
	γ-caprolactone	Poly(ε-CL-*co*-γ-CL)		
	Trimethylene carbonate	Poly(ε-CL-*co*-TMC)	Tin octanoate (SnOct_2_ )	[90,91]
			Methanesulfonic acid	[92]
	Carbonate, 2-allyloxymethyl-2-ethyl-trimethylene (AOMEC)	Poly(ε-CL-*co*- AOMEC)	1,5,7-triazabicyclo [4.4.0] dec-5-ene(TBD)	[93]
	PEG	Poly(ε-CL-*co*-EG)	PEG(NH_2_ )_8_/octoate, tin (SnOct_2_ )	[94]
			Tin octanoate (SnOct_2_ )	[51]
	Lactide	Poly(LA-*co*-ε-CL)	mPEG/octanoate, tin (SnOct_2_)	[95]
			1,4-benzenedimethanol / octoate, tin(SnOct_2_ )	[96]
	Methyl-δ-glucopyranoside	Oligo(MGP-*co*-ε-CL)	Immozyme CALB	[97]
	–	Oligo-ε-caprolactone	Tin octanoate (SnOct_2_ )/Boltorn H2004	[98]
			CALB	[19]
	Acetic acid 5-acetoxy-6-oxotetrahydropyran-2-yl methyl ester	Poly(AOTME-*co*-ε-CL)	Tin butoxide (Sn(Obu)_2_ )	[99]
	5-hydroxymethyl-2-furancarboxylic acid	Oligo(HMFA-*co*-ε-CL)	CALB	[100]
	Polyhexamethylene guanidine (PHMG)	Poly(HMG-*co*-ε-CL)	PHMG	[52]
	γ-(carbamic acid benzyl ester)-ε-caprolactone (CABCL)	Poly(γ-CABCL-*co*-ε-CL)	mPEG / octanoate, tin (SnOct_2_)	[101]
δ-decalactone	PEG	Poly(EG-*co*-δ-DL)	PEG/1,5,7-triazabicyclo [4.4.0] dec-5-ene (TBD)	[61]
	mPEG	Poly(mEG-*co*-δ-DL)	mPEG/1,5,7-triazabicyclo [4.4.0] dec-5-ene (TBD)	
	–	Poly-δ-decalactone	triazabicyclo [4.4.0] dec-5-ene (TBD) / 1,4-benzenedimethanol (BDM)	[102]
ε-decalactone	ω-pentadecalactone	Poly(ω-PDL-*co*-ε-DL)	Triphenyl bismuth (Ph_3_ Bi)	[103]
	–	Poly-ε-decalactone	triazabicyclo [4.4.0] dec-5-ene (TBD)/benzyl alcohol	[104]
			Tin octanoate (SnOct_2_)/Benzyl alcohol	[104]
			InCl_3_ / Triethylamine (NEt_3_ ) / Benzyl alcohol	[105]
			InCl_3_ / Triethylamine (NEt_3_ ) / benzyl amine	[105]
	Lactide	Poly(LA-*co*-ε-DL)	Tin octanoate (SnOct_2_ ) / 1,4-butanediol	[104]
			Tin octanoate (SnOct_2_ ) / 1,4-benzenedimethanol (BDM)	[106]
	ε-caprolactone	Poly(ε-CL-*co*-ε-DL)	InCl_3_ / Dimethoxybenzyl Alcohol (DMBA), Triethylamine (NEt_3_)	[105]
			Tin octanoate (SnOct_2_)/1,4-benzenedimethanol (BDM)	[62]
	mPEG	Poly(mEG-*co*-ε-DL)	mPEG	[63]

## Data Availability

Data sharing not applicable.
